# Association between NAD^+^ levels and anaemia among women in community‐based study

**DOI:** 10.1111/jcmm.17281

**Published:** 2022-04-06

**Authors:** Fan Yang, Xuguang Zhang, Feifei Hu, Ye Yu, Lei Luo, Xuan Deng, Yuzheng Zhao, Bo Pan, Jinping Zheng, Yugang Qiu, Jun Guo, Feng Xiao, Xiaomei Xie, Zhenyu Ju, Yong Zhou

**Affiliations:** ^1^ Institute of Aging and Regenerative Medicine The First Affiliated Hospital of Jinan University Jinan University Guangzhou China; ^2^ Science and Technology Centre By‐Health Co. Ltd. Guangzhou China; ^3^ Clinical Research Institute Shanghai General Hospital Shanghai Jiao Tong University School of Medicine Shanghai China; ^4^ Administrative Office Total Quality Management Office Total Quality Management Institute Shanghai General Hospital Shanghai Jiao Tong University School of Medicine Shanghai China; ^5^ 47860 State Key Laboratory of Bioreactor Engineering Shanghai Collaborative Innovation Center for Biomanufacturing Technology Optogenetics & Synthetic Biology Interdisciplinary Research Center Research Unit of Chinese Academy of Medical Sciences East China University of Science and Technology Shanghai China; ^6^ Department of Auricular Reconstruction Plastic Surgery Hospital Peking Union Medical College and Chinese Academy of Medical Science Beijing China; ^7^ 74652 Department of Public Health and Preventive Medicine Changzhi Medical College Changzhi China; ^8^ 372527 School of Rehabilitation Medicine Weifang Medical University Weifang China; ^9^ Tangshan Gem Flower Hospital Tangshan China

**Keywords:** cardiovascular diseases, hemoglobin, Nicotinamide adenine dinucleotide

## Abstract

Nicotinamide adenine dinucleotide (NAD^+^) level is the protective factor of cardiovascular diseases (CVDs). In addition, anaemia is a risk factor of adverse cardiovascular outcomes in women. However, there are limited data about the association between NAD^+^ and anaemia. The aim of this study was to evaluate association of NAD^+^ with anaemia among women. A total of 727 females from Jidong community were included in the current analysis. NAD^+^ levels were tested by the cycling assay and HPLC assay using whole blood samples. Anaemia was determined by haemoglobin (Hb) concentration, and the subtypes of anaemia were further defined according to mean corpuscular volume (MCV) in blood. Multivariable logistic analysis was used to analyse the association between NAD^+^ levels and anaemia or its subtypes. The mean age of recruited subjects was 42.7 years. The proportion of anaemia by NAD^+^ levels quartiles were 19.7% (35/178), 4.8% (9/189), 3.4% (6/178) and 2.7% (5/182). Haematological parameters including haemoglobin (Hb), mean corpuscular volume (MCV), mean corpuscular haemoglobin (MCH), mean corpuscular haemoglobin concentration (MCHC) and red blood count (RBC) increased over NAD^+^ quartiles. Red cell volume distribution width (RDW) decreased over NAD^+^ quartiles. Compared with the lowest quartile of NAD^+^ levels (<27.6μM), the adjusted odds ratios with 95% confidence intervals of the top quartile were 0.15 (0.06–0.41) for anaemia, 0.05 (0.01–0.36) for microcytic anaemia and 0.37 (0.10–1.36) for normocytic anaemia respectively. Higher NAD^+^ levels were significantly associated with lower prevalence of anaemia among women, especially microcytic anaemia and normocytic anaemia. Haematological parameters might serve as a predictor of the blood NAD^+^ levels.

## 1 INTRODUCTION

Nicotinamide adenine dinucleotide (NAD^+^) is a pivotal metabolite with a wide range of roles in cell survival, mitochondrial homeostasis, cellular bioenergetics, adaptive stress responses and genomic stability.^1^ NAD^+^ is involved in over 500 enzymatic reactions in regulating almost all major biological processes.^2^ Meanwhile, NAD^+^ is also a co‐substrate of regulatory enzymes, including sirtuins (SIRTs), poly (ADP‐ribose) polymerases (PARPs) and cyclic ADPR (cADPR) synthetases.^3^ The association of NAD^+^ levels with cardiovascular diseases (CVDs) including endothelial, atherosclerosis and heart failure has been reported by a few studies.^4^, ^5^, ^6^, ^7^ Loss of NAD^+^ contents are implicated in the pathogenesis of multiple types of CVDs, and boosting NAD^+^ levels seems to be a strongly protective role of CVDs.^8^ Moreover, NAD precursors have been suggested to delay the process of vascular aging and increase the span of cardiovascular health.^9^

Anaemia is a worldwide health problem in the world,^10^, ^11^, ^12^, ^13^ particularly so in developing countries with children and women as the most affected population groups.^14^ Approximately 1.9 billion individuals worldwide suffer from anaemia, which is nearly one‐quarter of the global population in 2013.^15^ The World Health Organization (WHO) estimated that 32.4 million pregnant women and 496.3 million non‐pregnant women were anaemic across the world in 2011.^16^ In addition, severe anaemia in pregnant and postnatal women strongly and independently contributes to maternal death.^17^ Managing anaemia is one of the global health goals.^13^ In China, whose population accounts for more than 18% of the world, the prevalence of anaemia is about 15.0% according to the fifth Chinese National Nutrition and Health Survey (CNNHS 2010–2012) and the anaemia prevalence for Chinese rural reproductive age women was 24.8% in 2012.^18^, ^19^Anaemia was defined by WHO as a lower haemoglobin (Hb) content than normal in whole blood.^20^ In addition, lower Hb is significantly and independently associated with adverse cardiovascular outcomes in women.^21^ Anaemia is significantly correlated with severe complications of CVDs including stroke, arrhythmias and thromboembolics in the general population. It is also an independent predictor of cardiovascular mortalities.^22^, ^23^, ^24^ NAD^+^ levels and anaemia are the protective factor and risk factor of CVDs respectively. However, the research on the associations between NAD^+^ contents in whole blood and subtypes of anaemia is still in scarcity. We assumed that NAD^+^ levels would be negatively correlated with anaemia. In our current study, we sought to understand the relationship of NAD^+^ levels with the anaemia and explore the associations of different types of anaemia with NAD^+^ levels.

## 2 METHOD

### 2.1 Study design and population

**FIGURE 1:**
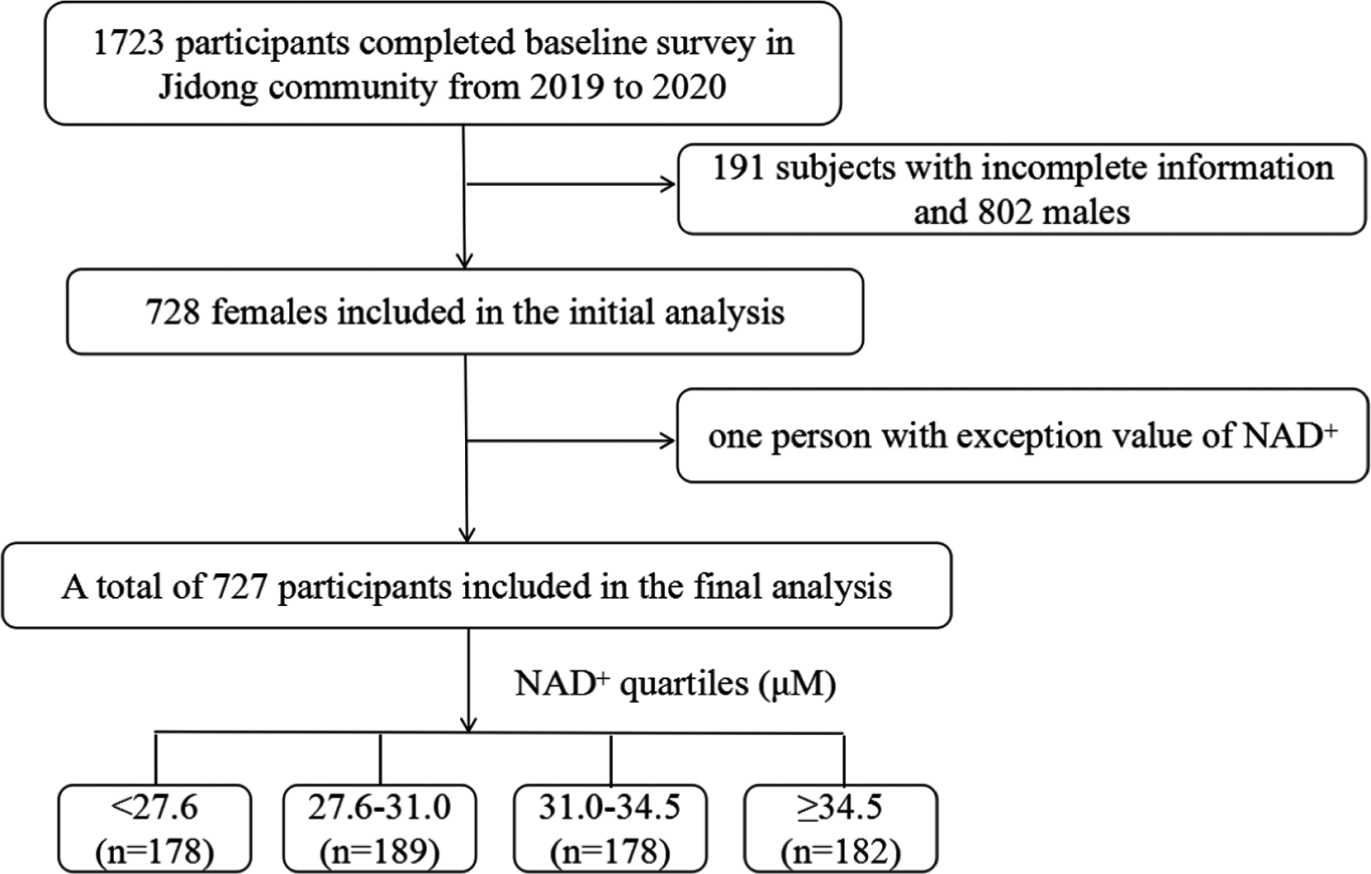
Flow chart of this study

The population in this community‐based study was from Jidong community in Tangshan City, Hebei Province, China. From 2019 to 2020, a total of 1723 participants were originally recruited into the study. We excluded 802 males and 191 participants with incomplete information. Finally, 727 females were included in the final analysis (Figure 1). All participants gave written informed consent, and the study was conducted according to the guidelines of the Helsinki Declaration.

### 2.2 Data collection

In this study, basic information of subjects was obtained from standardized questionnaires, laboratory tests and clinical examinations.^25^ Face‐to‐face interviews were performed by well‐trained examiners. Information on demographic characteristics including age, income and education level was collected by standardized questionnaires. The average monthly income was divided into ‘≤¥3000’, or ‘>¥3000’. Education levels were categorized as ‘Middle school or below’ or ‘collegeor above’. Body mass index (BMI) was categorized as ‘<18.5 kg/m^2’^, ‘18.5–23.9 kg/m^2’^, ‘24.0–27.9 kg/m^2’^ and ‘>28.0 kg/m^2’^. Previous history of hyperlipidaemia, hypertension and diabetes mellitus was recorded directly by self‐reports of participants.

### 2.3 Measurement of NAD^+^ levels

Blood samples were collected from the large antecubital veins after overnight fasting. All blood samples were stored in vacuum tubes containing EDTA (ethylene diamine tetraacetic acid), and NAD^+^ levels were determined by the cycling assay and LC‐MS/MS analysis in the laboratories.^26^, ^27^, ^28^, ^29^ (see Supplementary Material 1 and Figure S1).

NAD^+^ levels were stratified into 4 categories: Q1 (<27.6), Q2 (27.6–31.0), Q3 (31.0–34.5) and Q4 (≥34.5), which were based on the quartiles of NAD^+^ levels. In addition, the participants in Q2, Q3 and Q4 were grouped into Q2‐4, whose NAD^+^ level was in 2th‐4th NAD^+^ quartile (25th percentile‐100th percentile).

### 2.4 Determination of haematology parameters

Haematology parameters including haemoglobin (Hb), mean corpuscular volume (MCV), mean corpuscular haemoglobin (MCH), mean corpuscular haemoglobin concentration (MCHC), red cell distribution width (RDW) and red blood count (RBC) were measured by autoanalyzer (Hitachi 747; Hitachi,) in the central laboratory of the Staff Hospital of the Jidong Oilfield.

### 2.5 Diagnosis of anaemia

According to World Health Organization (WHO), anaemia was defined as Hb concentration lower than 120 mg/dl for women.^20^ According to MCV, anaemia was further classified into three types: microcytic anaemia if MCV was lower than 80 fl, normocytic anaemia if MCV was from 80 to 100 fl, and macrocytic anaemia if MCV was higher than 100 fl.^30^

### 2.6 Statistical analysis

The normality distributions of continuous variables were evaluated by the Kolmogorov–Smirnov test. Continuous variables are expressed as the mean ± standard deviation (SD) and were compared using one‐way ANOVA or *t*‐test, as appropriate. Categorical variables are presented as proportions and frequencies and were compared by chi‐squared tests. Multivariable logistic regression models were used to assess the association between NAD^+^ quartiles and anaemia or its different types. We adjusted 4 covariates which were thought to be potential confounder of the risk factors for anaemia: age, BMI, UA and RBC. All statistical tests were 2‐sided, and *p* values of less than 0.05 were considered to be significant. Statistical analyses were conducted with SAS software, version 9.4 (SAS Institute Inc.,).

## 3 RESULTS

### 3.1 Baseline Characteristics in eligible participants

Baseline characteristics of participants according to NAD^+^ quartiles are summarized in Table 1. Of 727 females finally included, the mean age was 42.7 years and there were only 6 smokers and 5 drinkers. BMI distributions were different among the quartiles of NAD^+^. The levels of UA increased along with the levels of NAD^+^. Analysis of haematological parameters in different NAD^+^ quartiles is also presented in Table 1. The levels of Hb, MCV, MCH, MCHC and RBC increased along with the quartiles of NAD^+^, while the levels of RDW decreased along with the quartiles of NAD^+^. Age, income, education level, history of smoke, history of drink, salt intake, eGFR, history of hyperlipidaemia, hypertension and diabetes were not significantly different among the quartiles. Baseline characteristics of male and females has been provided. (see Table S2).

**TABLE 1:** Baseline characteristics of participants according to NAD^+^ quartiles

Characteristics	Overall (*n* = 727)	Q1 (<27.6) (*n* = 178)	Q2 (27.6–31.0) (*n* = 189)	Q3 (31.0–34.5) (*n* = 178)	Q4 (≥34.5) (*n* = 182)	*p* value
Age (years)	42.7 ± 11.3	41.4 ± 9.3	43.5 ± 12.4	42.9 ± 11.5	42.9 ± 11.7	0.33
Income,¥/month (*n*,%)						0.33
≤¥3000	52 (7.8)	8 (5.0)	13 (7.7)	17 (10.6)	14 (8.1)	
>¥3000	613 (92.2)	152 (95.0)	157 (92.4)	177 (89.4)	160 (92.0)	
Education level (*n*,%)						0.57
Middle school or below	254 (34.9)	63 (35.4)	73 (38.6)	60 (33.7)	58 (31.9)	
College or above	473 (65.1)	115 (64.6)	116 (61.4)	118 (66.3)	124 (68.1)	
Body mass index (kg/m^2^)						<0.05
<18.5	99 (13.6)	23 (12.9)	25 (13.2)	29 (16.3)	22 (12.1)	
18.5–23.9	395 (54.3)	97 (54.5)	108 (57.1)	98 (55.1)	92 (50.6)	
24.0–27.9	178 (24.5)	53 (29.8)	43 (22.8)	31 (17.4)	51 (28.0)	
≥28.0	55 (7.6)	5 (2.8)	13 (6.9)	20 (11.2)	17 (9.3)	
Smoking (*n*,%)	6 (0.9)	0 (0.0)	3 (1.8)	2 (1.2)	1 (0.6)	0.43
Drinking (*n*,%)	5 (0.8)	2 (1.3)	0 (0.0)	2 (1.2)	1 (0.6)	0.46
Salt intake (*n*,%)						0.49
Low	218 (32.8)	55 (34.4)	53 (31.2)	49 (30.4)	61 (35.1)	
Medium	370 (55.6)	81 (50.6)	101 (59.4)	96 (59.6)	92 (52.9)	
High	77 (11.6)	24 (15.0)	16 (9.4)	16 (9.9)	21 (12.1)	
Hyperlipidaemia (*n*,%)	256 (38.4)	56 (35.0)	59 (34.7)	60 (37.0)	81 (46.6)	0.08
Hypertesion (*n*,%)	107 (16.1)	19 (11.9)	26 (15.3)	26 (16.1)	36 (20.7)	0.18
Diabetes (*n*,%)	35 (5.3)	7 (4.4)	6 (3.5)	8 (4.9)	14 (8.1)	0.26
eGFR (mL/min/1.73m^2^)	121.4 ± 24.3	121.0 ± 23.3	124.1 ± 24.4	118.7 ± 24.4	121.9 ± 24.7	0.28
UA (μmol/L)	289.9 ± 66.2	281.9 ± 61.4	283.1 ± 61.7	296.1 ± 68.8	298.6 ± 71.2	<0.05
Haematological parameters						
Hb (g/L)	136.0 ± 12.1	130.1 ± 15.6	135.6 ± 11.0	138.4 ± 9.4	139.8 ± 9.2	<0.0001
MCV (fL)	91.9 ± 6.4	89.9 ± 8.7	91.6 ± 6.5	93.1 ± 4.7	93.1 ± 4.2	<0.0001
MCH (pg)	30.9 ± 2.5	30.1 ± 3.4	30.6±2.5	31.4 ± 1.7	31.4 ± 1.5	<0.0001
MCHC (g/L)	335.6 ± 8.8	334.2 ± 10.1	334.1 ± 8.4	337.0 ± 7.5	337.2 ± 8.4	<0.0001
RDW (%)	12.2 ± 1.5	12.6 ± 2.1	12.2 ± 1.6	12.0 ± 1.1	12.0 ± 1.0	<0.01
RBC (10^12/L)	4.4 ± 0.3	4.3 ± 0.4	4.4 ± 0.3	4.4 ± 0.3	4.5 ± 0.3	<0.01
PLT (10^9/L)	229.7 ± 57.8	235.6 ± 61.5	235.6 ± 58.9	219.5 ± 50.1	227.8 ± 59.1	<0.05
WBC (10^9/L)	6.3 ± 1.5	6.3 ± 1.6	6.3 ± 1.5	6.3 ± 1.5	6.4 ± 1.6	0.77

Abbreviations: BMI, body mass index; eGFR, estimated glomerular filtration rate; UA, uric acid; NAD^+^, nicotinamide adenine dinucleotide; Hb, haemoglobin; MCV, mean corpuscular volume; MCH, mean corpuscular; MCHC, mean corpuscular haemoglobin concentration; RDW, red cell volume distribution width; RBC, red blood cell; PLT, platelet count; WBC, white blood cell.

### 3.2 Association between NAD^+^ levels and anaemia

**FIGURE 2:**
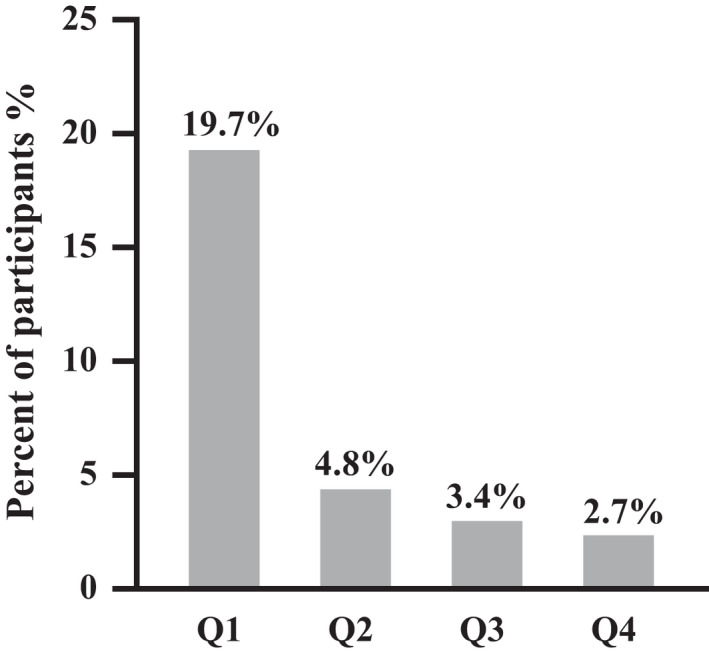
Proportions of anaemia status according to NAD^+^ quartiles

The anaemia occurred in 19.7%, 4.8%, 3.4% and 2.7% in each NAD^+^ quartile respectively (Figure 2). Crude and adjusted odds ratios (OR) with 95% confidence intervals (CI) of NAD^+^ levels for anaemia status are shown in Table 2. In general, lower NAD^+^ levels were associated with higher prevalence of anaemia. Compared with the first quartile of NAD^+^, proportion rates of anaemia in the 2th‐4th NAD^+^ quartile (25th percentile‐100th percentile) was also much lower. The NAD^+^ levels between anaemic and non‐anaemic group have been provided. (see Table S3).

**TABLE 2:** Association between quartiles of NAD^+^ levels and anaemia among women

NAD^+^ Quartiles	Subjects with anaemia (n,%)	Unadjusted OR (95% CI)	Adjusted OR (95% CI)	
Q1	35 (4.8)	1 (Reference)	1 (Reference)	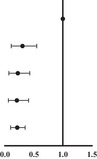
Q2	9 (1.2)	0.20 (0.10–0.44)	0.25 (0.11–0.55)	
Q3	6 (0.8)	0.14 (0.06–0.35)	0.17 (0.07–0.43)	
Q4	5 (0.7)	0.12 (0.04–0.30)	0.15 (0.06–0.41)	
Q2‐4	20 (2.8)	0.15 (0.09–0.28)	0.19 (0.10–0.35)	


Note

Covariates included age, BMI, UA and RBC.

Abbreviations: CI, confidence interval; OR, odds ratio.

### 3.3 Association between quartiles of NAD^+^ levels and types of anaemia

Rates of types of anaemia according to quartiles of NAD^+^ levels are presented in Table 3. The proportion of microcytic anaemia and normocytic anaemia was 4.1% and 3.5%. Both of them decreased by NAD^+^ quartiles. The proportion of macrocytic anaemia was just 0.1% (*N* = 2), so macrocytic anaemia was not further analysed in the logistic regression model due to few events. As shown in Table 4, the prevalence decreased with the higher NAD^+^ levels in microcytic anaemia and normocytic anaemia respectively; compared with the first quartiles of NAD^+^, the adjusted ORs and 95% CI of the fourth NAD^+^ quartile were 0.05 (0.01–0.36) for microcytic anaemia and 0.37 (0.10–1.36) for normocytic anaemia, and the adjusted ORs and 95% CI of the 2th~4th NAD^+^ quartile (25th percentile‐100th percentile) were 0.16 (0.07–0.36) for microcytic anaemia and 0.20 (0.07–0.56) for normocytic anaemia. The haematological parameters in each type of anaemia have been provided. (see Table S1).

**TABLE 3:** Rates of types of anaemia according to quartiles of NAD^+^ levels among women

		NAD^+^ Quartiles, μM				
Type of anaemia	Overall	Q1 (<27.6)	Q2 (27.6–31.0)	Q3 (31.0–34.5)	Q4 (≥34.5)	*p* value
microcytic anaemia	29 (4.1)	18 (2.6)	7 (1.0)	3 (0.4)	1 (0.1)	<0.0001
normocytic anaemia	24 (3.5)	15 (2.2)	2 (0.3)	3 (0.4)	4 (0.6)	<0.001

**TABLE 4:** Association between quartiles of NAD^+^ levels and types of anaemia among women

Type of anaemia	Unadjusted OR (95% CI)	Adjusted OR (95% CI)	
Microcytic anaemia			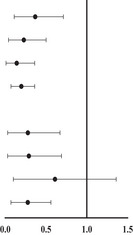
Q2 VS Q1	0.31 (0.13–0.76)	0.28 (0.11–0.71)	
Q3 VS Q1	0.14 (0.04–0.48)	0.14 (0.04–0.50)	
Q4 VS Q1	0.05 (0.01–0.34)	0.05 (0.01–0.36)	
Q2‐4 VS Q1	0.17 (0.08–0.36)	0.16 (0.07–0.36)	
Normocytic anaemia			
Q2 VS Q1	0.11 (0.02–0.47)	0.13 (0.03–0.67)	
Q3 VS Q1	0.17 (0.05–0.59)	0.15 (0.03–0.69)	
Q4 VS Q1	0.22 (0.07–0.66)	0.37 (0.10–1.36)	
Q2‐4 VS Q1	0.16 (0.07–0.38)	0.20 (0.07–0.56)	


Note

Covariates included age, BMI, UA and RBC.

Abbreviations: CI, confidence interval; OR, odds ratio.

## 4 DISCUSSION

In this community‐based study, individuals with the low NAD^+^ quartile were associated with high risk of anaemia among women. Anaemia subtype analysis in our study showed that this association was also evident in microcytic anaemia and normocytic anaemia among women. Besides, we observed a positive association between NAD^+^ levels and haematological parameters including Hb, MCV, MCH, MCHC and RBC. Our results provide evidence for the relationship between NAD^+^ levels and anaemia among women.

Most of the existing studies focused on the association between NAD^+^ contents in RBCs and sickle cell disease (SCD). Studies about the association between SCD and NAD^+^ contents in RBCs are controversial. Sickle RBCs had an increased NAD^+^ content, and this increase in NAD^+^ may be the reason of adverse metabolic consequences in sickle RBCs.^31^ However, a study reported that the levels of NAD^+^ in sickle RBCs were similar to the levels in normal RBCs.^32^ As reported, among patients with anaemia, 50%–80% are iron deficiency anaemia (IDA) which is highly prevalent among women throughout their lives.^33^ A study in rhesus monkey found that the NAD metabolites were similar between IDA and control groups, and NAD pathway components nearly doubled after the treatment of IDA.^34^ Another study in male chicks found that iron deficiency reduced the utilization of tryptophan with which de novo biosynthesis of NAD starts.^35^, ^36^ Haemolytic anaemia could be induced by medications along with other causes, whose late diagnosis could be fatal.^37^ Nicotinamide mononucleotide adenylyltransferase 3 (Nmnat3) is considered a NAD synthesis enzyme involved in de novo and salvage pathways. Deficiency of Nmnat3 in mice can cause haemolytic anaemia.^38^ In our study, the anaemia subtypes distinguished by pathogeny like IDA and haemolytic anaemia could not be judged and anaemia was classified into three subtypes by MCV. NAD^+^ levels were detected in whole blood instead of just in RBCs. We found that low NAD^+^ quartile was associated with high risk of anaemia among women. The prevalence of microcytic anaemia and normocytic anaemia decreased with increase of NAD^+^ level. The specific impact of NAD^+^ levels on anaemia warrants further research.

Elevated Hb was related to Sirtuin 1 (SIRT1) levels, which was the activation of NAD‐dependent deacetylase.^39^ A study in mice found that replenishing NAD had a positive effect on the most primitive blood stem cells and protected patients from haematological failure.^40^ These findings indicated that NAD^+^ might be a protective factor of anaemia and also provided a possible theoretical support for our findings. In our study, there were positive associations between NAD^+^ levels and haematological parameters including Hb, MCV, MCH, MCHC and RBC. Our result implied that, NAD^+^ might be a new indicator for anaemia among women, especially in microcytic anaemia and normocytic anaemia.

The study has several potential limitations. First, we were unable to determine subtype of anaemia according to the pathogeny due to the limited data. Thus, the associations of anaemia subtypes according to the pathogeny with NAD^+^ levels need to be further investigated in a subsequent study. Second, the cross‐sectional study made it difficult to infer the causal effect relationship between anaemia and NAD^+^ contents. Third, the participants were mainly from an urban city in North China; therefore, the findings might not be generalized to other ethnics and male. Finally, given the characteristics of observational study, there might be some unmeasured or residual confounding effects that could not be adjusted.

The study aim was to investigate the association between NAD^+^ level and the prevalence of anaemia subtypes distinguished by MCV and the correlation of NAD^+^ with haematological parameters among women. Due to the limited data, the association between NAD^+^ level and specific anaemia subtypes according to the pathogeny could not be analysis. Whether NAD^+^ is involved in the occurrence and development of anaemia, has core effect or just was accompanied by appearance, still need to be studied in future research.

In summary, the high NAD^+^ level in whole blood was associated with a low prevalence of anaemia among women, especially microcytic anaemia. Besides, haematological parameters including Hb, MCV, MCH, MCHC and RBC were positively associated with NAD^+^ contents. Haematological parameters might serve as a predictor for lack of NAD^+^ in whole blood among women.

## CONFLICT OF INTEREST

The authors declare no competing interests.

## AUTHOR CONTRIBUTIONS

**Yong Zhou:** Funding acquisition (equal); Project administration (lead). **Fan Yang:** Data curation (lead); Formal analysis (equal); Methodology (supporting); Writing – original draft (supporting); Writing – review & editing (supporting). **Xuguang Zhang:** Data curation (supporting); Formal analysis (supporting); Methodology (supporting); Project administration (supporting); Writing – review & editing (supporting). **Feifei Hu:** Data curation (equal); Formal analysis (equal); Methodology (supporting); Project administration (supporting); Writing – original draft (lead); Writing – review & editing (lead). **Ye Yu:** Data curation (equal); Writing – review & editing (equal). **Lei Luo:** Data curation (supporting); Formal analysis (supporting); Methodology (supporting); Project administration (supporting). **Xuan Deng:** Data curation (supporting); Methodology (supporting); Project administration (supporting). **Yuzheng Zhao:** Data curation (supporting); Methodology (supporting); Project administration (supporting). **Bo Pan:** Data curation (supporting); Methodology (supporting); Project administration (supporting). **Jin‐ping Zheng:** Data curation (supporting); Methodology (supporting); Project administration (supporting). **Yugang Qiu:** Data curation (supporting); Methodology (supporting); Project administration (supporting). **Feng Xiao:** Data curation (supporting); Funding acquisition (supporting); Methodology (supporting); Project administration (supporting). **Zhenyu Ju:** Data curation (equal); Formal analysis (equal); Funding acquisition (equal); Methodology (equal); Project administration (equal); Writing – review & editing (equal). **Jun Guo:** Methodology (supporting); Validation (supporting). **Xiaomei Xie:** Data curation (supporting); Investigation (supporting).

## Supporting information

Supplementary Material
