# Supplementary material for: Association between NAD+ levels and anaemia among women in community‐based study
Source: J Cell Mol Med. 2022 Apr 6;26(9):2698–705. doi: 10.1111/jcmm.17281 (PMC9077291; doi:10.1111/jcmm.17281)
Supplement: Supplementary file 1 — Supplementary Material [file JCMM-26-2698-s001.docx]

Supplementary material 1. Measurement of NAD^+^ levels

Blood samples were collected from the large antecubital veins after overnight fasting. All blood samples were stored in vacuum tubes containing EDTA (ethylene diamine tetraacetic acid), and NAD^+^ levels were determined by the cycling assay and LC-MS/MS analysis in the laboratories.

For cycling assay, blood samples were extracted with 0.5M HClO_4,_ then centrifuged at 10,000xg for 15 min. Organic solvent (1,1,2-trichloro-1,2,2-trifluoroethane : Trioctylamine = 3:1) was added to remove HClO_4_ at a ratio of 2:1. Carefully removed the top aqueous layer containing the NAD^+^ and added 1 M Tris to adjust the pH to 8.0. 100ul supernatants were mixed with 100ul reaction medium containing CCK-8, 13 units/ml alcohol dehydrogenase, 100 mM nicotinamide, 5.7% ethanol in 61 mM Gly-Gly buffer (pH 7.4). Samples were mixed in a 96-well plate samples at room temperature. The A450 nm was determined immediately after 30 min, and results were calibrated with NAD^+^ standards. Total NAD^+^ were quantified using a plate reader. Because of experiment variation in sample processing, samples were processed in parallel. The normalized values from each experiment were used to obtain the presented average values.

For LC-MS/MS analysis, stock solution preparation: NAD^+^ (1 mM in water) and ^13^C_5_-NAD^+^ (IS, 1mM in water) were stored at -80℃.Working standards were prepared by diluting above stocks in water containing 500 nM internal standard. Blood samples: Samples were diluted in HClO_4_ containing 500 nM of internal standard. Samples were transferred to sample vials for immediate analysis. NAD^+^ analysis in blood samples was carried out with an Agilent 1260 couples to a QTRAP@ 4500 mass spectrometer (AB SCIEX) equipped with an electrospray ionization source. The aqueous mobile phase was water with ammonium acetate (A) and the organic mobile phase was methanol (B) with a constant flow rate of 0.4 ml/min and a total run time of 11 min. The elution was initiated at 96% A and held for 1.5 min, then B was increased to 100% for 2.5 min, and returned to initial conditions in 4.5 min. Auto-sampler temperature was 22-25℃ and sample injection volume was 5 μl and NAD^+^ was eluted at 3.19 min. Detection of NAD^+^ was accomplished using the mass spectrometer in negative ESI mode using capillary voltage 4.5 kV, source temp 400℃, desolvation gas I flow 55 psi and gas II flow 50 psi. NAD^+^ quantitation was carried using the multiple reaction monitoring (MRN) transition m/z 662.1>540.0 for NAD^+^ and m/z 667.1>545.1 for the internal standard (^13^C_5_-NAD^+^). Data was acquired and analyzed by SCIEX v1.6.3 software. The reliability of the NAD^+^ levels obtained with the cycling assay was verified by comparing the data with those determined by conventional LC-MS/MS (Figure S1).

Supplementary Figure 1. Correlation between Enzymatic Assay and LC-MS/MS


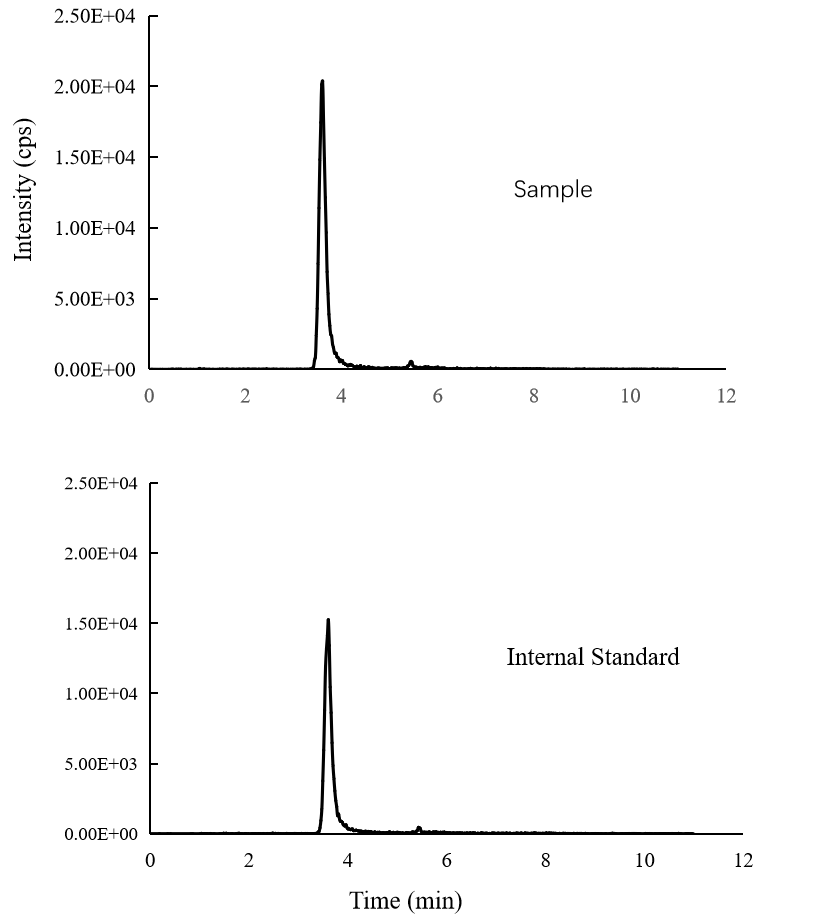
A

B

C

| Regression equation | SE.EST | Mean bias(±SD) (uM) | Mean ratio (±SD) | Range of ratios |
| --- | --- | --- | --- | --- |
| Enzymatic Assay =0.933*(LC/MS/MS)+2.9086 | 1.43 | 0.47±1.42 | 0.99±0.02 | 0.90-1.08 |

Correlation between Enzymatic Assay and LC-MS/MS. (A) The representative chromatograms of NAD^+^ and ^13^C_5_-NAD^+^ in human blood. (B-C) Blood concentrations measured by Enzymatic Assay compared with the reference method (LC/MS/MS) in 31 adult.

Supplementary Table 1. Hematological parameters in each type of anemia

| Hematological parameters | microcytic anemia  (n=29) | normocytic anemia  (n=24) | macrocytic anemia  (n=2) | *P* value |
| --- | --- | --- | --- | --- |
| Hb (g/L) | 100.0 ± 12.8 | 112.7 ± 7.0 | 117.0 ± 0.0 | 0.0001 |
| MCV (fL) | 71.3 ± 6.2 | 88.4 ± 5.4 | 111.0 ± 7.1 | <0.0001 |
| MCH (pg) | 22.6 ± 2.8 | 29.5 ± 2.0 | 36.8 ± 2.1 | <0.0001 |
| MCHC (g/L) | 316.1 ± 13.7 | 334.2 ± 9.1 | 331.3 ± 2.1 | <0.0001 |
| RDW (%) | 16.4 ± 2.3 | 13.6 ± 1.6 | 12.7 ± 1.5 | <0.0001 |
| RBC (10^12/L) | 4.4 ± 0.3 | 3.8 ± 0.3 | 3.2 ± 0.2 | <0.0001 |

Supplementary Table 2. Baseline characteristics of males and females

| Characteristics | Overall  (n=1528) | Women  (n=727) | Men  (n=801) | *P* value |
| --- | --- | --- | --- | --- |
| Age (years) | 43.2± 11.3 | 42.7 ± 11.3 | 43.6 ± 11.4 | 0.11 |
| Anemia(n,%) | 56 (3.7) | 55 (7.6) | 1 (0.1) | <0.0001 |
| Hematological parameters |  |  |  |  |
| Hb (g/L) | 149.2 ± 16.7 | 136.0 ± 12.1 | 161.2 ±10.0 | <0.0001 |
| MCV (fL) | 93.9 ±5.8 | 91.9 ± 6.4 | 94.5 ± 4.9 | <0.0001 |
| MCH (pg) | 31.4 ± 2.2 | 30.9 ±2.5 | 31.9 ± 1.8 | <0.0001 |
| MCHC (g/L) | 336.9 ± 8.0 | 335.6 ± 8.8 | 338.1 ± 7.1 | <0.0001 |
| RDW (%) | 12.0 ± 1.2 | 12.2 ± 1.5 | 11.9 ±0.8 | <0.0001 |
| RBC (10^12/L) | 4.8 ± 0.5 | 4.4 ± 0.3 | 5.1 ± 0.4 | <0.0001 |

Supplementary Table 3. NAD^+^ levels between anemic and non-anemic group

|  | NAD^+^ Quartiles, μM | | | |
| --- | --- | --- | --- | --- |
|  | Overall | Anemia  (n=55) | Non-anemia  (n=672) | *P* value |
| NAD^+^ (μM) | 31.3 ± 5.2 | 27.4 ± 4.3 | 31.6 ± 5.2 | <0.0001 |
| Body mass index (kg/m^2^) |  |  |  | 0.22 |
| <18.5 | 99 (13.6) | 11 (20.0) | 88 (13.1) |  |
| 18.5–23.9 | 395 (54.3) | 30 (54.6) | 365 (54.3) |  |
| 24.0-27.9 | 178 (24.5) | 13 (23.6) | 165 (24.6) |  |
| ≥28.0 | 55 (7.6) | 1 (1.8) | 54 (8.0) |  |
